# Supplementary material for: Prevalence of bovine tuberculosis and characterization of the members of the Mycobacterium tuberculosis complex from slaughtered cattle in Rwanda
Source: PLoS Negl Trop Dis. 2022 Aug 3;16(8):e0009964. doi: 10.1371/journal.pntd.0009964 (PMC9377585; doi:10.1371/journal.pntd.0009964)
Supplement: Attachment — Submitted filename: 25.11.2021 (Comments to authors).docx [file pntd.0009964.s002.docx]

**Comments to authors**

**Manuscript title: ‘Prevalence of bovine tuberculosis and characterization of the members of the *Mycobacterium tuberculosis* complex from slaughtered cattle in Rwanda.**

This work aimed to determine the prevalence of bovine tuberculosis and distinguish amongst members of the *Mycobacterium tuberculosis* complex (MTBC) species. The study is first to confirm *Mycobacterium bovis*, *Mycobacterium tuberculosis* and non-tuberculous mycobacteria in Rwanda. Additional interesting finding from the study was that not all TB-like lesions observed resulted in the isolation of Mycobacterium species, highlighting the importance of laboratory confirmation of the causative agent to obtain accurate diagnosis. Considering the moderate sample size analyzed, authors’ recommended future longitudinal studies which will consider a good sample size per district in that country. It will also be worthwhile to genotype MTBC identified to determine the genetic diversity of the strains circulating in Rwanda for epidemiological studies.

**Specific comments are as follows:-**

**Study area**

Line 96…Fig 1: Maps of (A) Africa with a red rectangle indicating the location of Rwanda; (B) Rwanda with provinces and districts and red stars show the locations of abattoirs visited in this study.

**Study design and sample size**

Line 117… P is an expected of 0.5% based on a…..Please rephrase. It is not clear what this means.

Line 120…cattle to include and respect the systematic sampling of 10 cattle per each of the 30 districts..’Per’ means ‘for each’. Please rephrase.

**Sampling procedure and tissue collection**

Line 130-133….. Upon decapitation, the marked head was inspected, and medial and lateral retropharyngeal lymph nodes and lingual and palatine tonsils were aseptically collected into the same sterile 50 ml tube (pooled) and stored at - 20ºC until processing.

**Culture and detection of acid fast bacilli (AFB)**

Line 137…. Please rephrase to say..’ an aliquot of each pool of tissue samples was kept at -20ºC…

**Results**

Line 216…Out of the 300 sample sets… replace ‘sets’ with ‘pools’

Line 218….300 samples… add pools (i.e. 300 sample pools). Implement throughout the document.

Fig 2: Agarose Gel Electrophoresis of the *Mycobacterium tuberculosis* complex differential PCR assay. Lanes 4-5 are missing on the gel picture (labeling), automatically affecting all labeling. For example, lane 11 is supposed to be a negative control according to the figure legend, however, there is application (PCR products). Lane 12 is supposed to be a positive control (M. tuberculosis), but there is no amplification.

**Discussion**

Line 248-249…..Although the prevalence of bTB (1.7%, 5/300) was low, the identification of *M. bovis* and 249 *M. tuberculosis* with rifampicin resistance in cattle indicates the cross-transmission from humans 250 to cattle…….This statement will only be mostly applicable to the identification of *M. tuberculosis* as it is the main causative agent of tuberculosis in human, but not really *M. bovis* (though humans can be diagnosed with zoonotic TB).

**Conclusion**

Line 328. This study demonstrated that bTB is prevalent in Rwanda at low prevalence. Please replace prevalent with ‘ present’.

**General comment**

Some line numbers in the results section are repeated in the discussion section (i.e. Line 242-255).
