# Supplementary material for: Prevalence of bovine tuberculosis and characterization of the members of the Mycobacterium tuberculosis complex from slaughtered cattle in Rwanda
Source: PLoS Negl Trop Dis. 2022 Aug 3;16(8):e0009964. doi: 10.1371/journal.pntd.0009964 (PMC9377585; doi:10.1371/journal.pntd.0009964)
Supplement: Attachment — Submitted filename: response to reviewers - 2-.docx [file pntd.0009964.s003.docx]

**Responses to Reviewers**

**Manuscript title: ‘Prevalence of bovine tuberculosis and characterization of the members of the *Mycobacterium tuberculosis* complex from slaughtered cattle in Rwanda.**

The authors would like to thank the journal, editor and reviewers for their time and inputs. ***The corresponding author apologizes for the delay to respond due to the sickness.*** Comments were addressed.

**Specific comments are as follows:-**

**Comment 1:**

Figure [1]: please (a) provide a direct link to the base layer of the map used and ensure this is also included in the figure legend; (b) provide a link to the terms of use / license information for the base layer. We cannot publish proprietary or copyrighted maps (e.g. Google Maps, Mapquest) and the terms of use for your map base layer must be compatible with our CC-BY 4.0 license.

**Response:**

Map (a) was removed from the manuscript due to lack of copyright.

Map (b) was produced in this study using the ArcGis Desktop 10.1 – 10.8.2 with concurrent use – and Education license of the University of Rwanda. The license was not provided for publication. The spatial data or shapefiles used were freely available from DIVA-GIS at [https://www.diva-gis.org/.](https://www.diva-gis.org/)

**Comment:**

1) When completing the data availability statement of the submission form, you indicated that " All relevant data are within the manuscript and its Supporting Information files". However, you also checked the following box: “Tick here if the URLs/accession numbers/DOIs will be available only after acceptance of the manuscript for publication so that we can ensure their inclusion before publication.” If this box was checked in error, and there is no additional data to deposit, please ensure this box is unticked when you resubmit.

**Response:**

The box was previously checked by error and this box was unticked at this submission

**Comment:**

2) Please upload the separate figure files in numerical order

**Response:**

Separate figure files were uploaded in numerical order.

3) Please ensure that all author names are spelled correctly in the manuscript file, including the use of a middle name/initials, and any special characters (accents, umlauts, etc.). Please place first/given names before last/family names. All authors must have a first and last name. We noticed that " Jean Bosco Ntivuguruzwa, Francis Babaman Kolo, Ivan Emil Mwikarago, Claude Semuto Ngabonziza" are spelled differently between the manuscript file and submission form. Please be aware that correct spelling and formatting of author names in the article file is vital for accurate indexing in PubMed.

**Response:**

The names of authors were harmonized accordingly.

**Comment:**

4) We do not publish any copyright or trademark symbols that usually accompany proprietary names, eg (R), (C), or TM (e.g. next to drug or reagent names). Please remove all instances of trademark/copyright symbols throughout the text, including MyTaqTM on line no 173.

**Response:**

Trademark symbols were removed.

**Comment:**

5) Please amend your detailed Financial Disclosure statement. This is published with the article, therefore should be completed in full sentences and contain the exact wording you wish to be published. i). State the initials, alongside each funding source, of each author to receive each grant.

**Response:**

This was checked and corrected.
