# Supplementary material for: Prevalence of bovine tuberculosis and characterization of the members of the Mycobacterium tuberculosis complex from slaughtered cattle in Rwanda
Source: PLoS Negl Trop Dis. 2022 Aug 3;16(8):e0009964. doi: 10.1371/journal.pntd.0009964 (PMC9377585; doi:10.1371/journal.pntd.0009964)
Supplement: Attachment — Submitted filename: Response to reviewers - June.docx [file pntd.0009964.s004.docx]

**Response to reviewers**

**June 23^rd^, 2022**

**Reviewer 1 and Reviewer 2**

**PLOS Neglected Tropical Disease**

**Re: revision of the manuscript PNTD-D-21-01588R1 - [EMID: 41bdd8507f9ad72b]**

Dear reviewers,

The authors would like to acknowledge your inputs. We thank you for reviewing our manuscript entitled “**Prevalence of bovine tuberculosis and characterization of the members of the *Mycobacterium tuberculosis* complex from slaughtered cattle in Rwanda**”. Your comments were very clear and useful to improve the quality of this manuscript. We have revised the manuscript considering all issues mentioned in your comments. Outlined below are the point-by-point responses.

**Reviewer #1**: Overall, the manuscript is well written and scientifically sound. The aim and objectives are clearly articulated and to the point. Well known statistical methods were used to determine the sample size and results analysis. The study was approved by relevant ethics committees.

**Response:** Thank you so much

**Reviewer #2**: (No Response)

**Response:** thank you so much

**Reviewer #1**: Overall, results were presented well. However, minor additions/clarity is needed in table 1 and figure 1.

**Response:** minor additions/clarity were made in results section. Thank you so much

**Reviewer #2**: The essential changes in the gel picture have been carried out and the response to the queries has been carried out satisfactorily.

**Response:** Thank you so much.

**Reviewer #1:** This is a cross sectional study that utilized a systematic sampling of cattle as well as mycobacterial species culture and molecular based techniques to determine the prevalence of bovine tuberculosis and characterize members of the isolated Mycobacterium tuberculosis complex (MTBC). The manuscript was well written and it is scientifically sound.

**Comments:-Introduction**

Line 65…. It will be worthwhile to mention other members of the MTBC that cause bovine tuberculosis besides M. bovis and M. caprae before mentioning that M. tuberculosis infection in cattle has been identified more recently in African countries….

**Response:** comment addressed as follows:

The disease is primarily caused by *Mycobacterium bovis* (1), however, other members of the MTBC have also been identified in diseased cattle such as *M. africanum* (2), *M. caprae* (3-5), *M. tuberculosis* (6, 7) and *M. orygis* (8). *Mycobacterium tuberculosis* infection in cattle has been identified more recently from African countries and is of concern (9-12)

**Conventional PCR**

Line 179-180…Authors should also mention the fragment sizes expected for the identification of M. tuberculosis….

**Response:** comment addressed as follows:

Primers amplified 1030 bp, 372 bp, 108 bp and 268 bp, and 172 bp and 235 bp fragments for the genus, MTBC, *M. bovis,* and *M. tuberculosis*, respectively. PCR products were analysed by electrophoresis using a 2% agarose gel stained with red gel nucleic acid stain and fragments were visualized under UV light.

**GeneXpert MTB/RIF assay**

Line 192-193… Cartridges were ‘installed’ into the Gene Xpert DX system… Please replace ‘installed’ with ‘loaded’….

**Response:** installed replaced by loaded

**Table 1**

Authors should make is clear which target gene sequence is present/absent in which mycobacterium species (i.e. RD4 is absent in M. bovis but present in M. tuberculosis etc…)

**Response:** comment was addressed as follows:

| **PCRs** | **Primer name** | **Nucleotide sequence (5’-----------3’)** | **Target** | **Gene or RD presence/ absence** | **Size (bp)** | **Tm (^o^C)** | **References** |
| --- | --- | --- | --- | --- | --- | --- | --- |
| **Multiplex 1** | Mycgen-F | AGA GTT TGA TCC TGG CTC AG | 16s rRNA | Gene is present in *M. bovis* and *M. tuberculosis* | 1030 | 62 | (13) |
|  | Mycgen-R | TGC ACA CAG GCC ACA AGG GA |  |  |  |  |  |
|  | TB1-F | GAA CAA TCC GGA GTT GAC AA | MPB 70 | Gene is present in *M. bovis* and *M. tuberculosis* | 372 |  |  |
|  | TB2-R | AGC ACG CTG TCA ATC ATG TA |  |  |  |  |  |
| **Multiplex 2*** | RD1-1 | AAGCGGTTGCCGCCGACCGACC |  | RD1 present in *M. bovis* and *M. tuberculosis* | 146 | 62 | (14) |
|  | RD1-2 | CTGGCTATATTCCTGGGCCCGG |  |  |  |  |  |
|  | RD1-3 | GAGGCGATCTGGCGGTTTGGGG |  |  |  |  |  |
|  | RD4-1 | ATG TGC GAG CTG AGC GAT G | Rv 1510 | RD4 absent in *M. bovis* but present in *M. tuberculosis* | 268 RD is absent | 62 | (15) |
|  | RD4-2 | TGT ACT ATG CTG ACC CAT GCG |  |  |  |  |  |
|  | RD4-3 | AAA GGA GCA CCA TCG TCC AC |  |  |  |  |  |
|  | RD9-1 | CAA GTT GCC GTT TCG AGC C | Rv 2073 | RD9 absent in *M. bovis* but present *M. tuberculosis* | 108 RD is absent | 62 | (14) |
|  | RD9-2 | CAA TGT TTG TTG CGC TGC |  |  |  |  |  |
|  | RD9-3 | GCT ACC CTC GAC CAA GTG TT |  |  |  |  |  |

**Figure 2**

Line 250… Authors should please indicate what was used as a negative control for quality control purposes.

**Response:** comment was addressed as follows: lane 251: sterile water was used as negative control

**Discussion**

Line 262. Remove ‘Therefore’ at the beginning of the sentence as this point does not relate to the previous one.

**Response:** Therefore was removed

**Reviewer #2**: Data has been represented in a better manner

Response: thank you

**Summary and General Comments**

Use this section to provide overall comments, discuss strengths/weaknesses of the study, novelty, significance, general execution and scholarship. You may also include additional comments for the author, including concerns about dual publication, research ethics, or publication ethics. If requesting major revision, please articulate the new experiments that are needed.

**Reviewer #1:** Indicated in the section above.

**Response:** the comments were addressed in the above section

**Reviewer #2**: Can be considered for publication

Response: thank you
